# Mindfulness Training for Primary Care for Portuguese-Speaking Immigrants: A Pilot Study

**DOI:** 10.3389/fpsyt.2021.664381

**Published:** 2021-09-09

**Authors:** Marcelo Trombka, Timothy B. Creedon, Marcelo Demarzo, Letícia T. Cuoco, Lydia Smith, Alexandra C. Oxnard, Alana T. Rozembaque, Marcio S. Hirayama, Natalia B. Moreno, Alexandra Comeau, Richa Gawande, Todd Griswold, Benjamin L. Cook, Neusa S. Rocha, Zev Schuman-Olivier

**Affiliations:** ^1^Postgraduate Program in Psychiatry and Behavioral Sciences, Federal University of Rio Grande do Sul (UFRGS), Porto Alegre, Brazil; ^2^Department of Psychiatry–Hospital de Clínicas de Porto Alegre, Porto Alegre, Brazil; ^3^Innovations and Interventions for Quality of Life Research Group, Porto Alegre, Brazil; ^4^Clinical Research Center–Hospital de Clínicas de Porto Alegre, Porto Alegre, Brazil; ^5^Cambridge Health Alliance, Center for Mindfulness and Compassion, Cambridge, MA, United States; ^6^Department of Psychiatry, Harvard Medical School, Boston, MA, United States; ^7^Cambridge Health Alliance, Health Equity Research Lab, Cambridge, MA, United States; ^8^Department of Preventive Medicine, Mente Aberta–Brazilian Center for Mindfulness and Health Promotion, Federal University of São Paulo (UNIFESP), São Paulo, Brazil; ^9^Mente Aberta—Brazilian Center for Mindfulness and Health Promotion, Federal University of São Paulo (UNIFESP), São Paulo, Brazil

**Keywords:** mindfulness, Portuguese, immigrants, depression, anxiety, self-regulation, health behavior, primary care

## Abstract

**Background:** Portuguese-speaking immigrants are a growing underserved population in the Unites States who experience high levels of psychological distress and increased vulnerability to mental health disorders such as depression and anxiety. Current evidence shows that mindfulness-based interventions (MBIs) are effective to promote physical and mental health among educated English speakers; nonetheless, the lack of diversity in the mindfulness literature is a considerable limitation. To our knowledge, the feasibility and acceptability of MBIs among Portuguese-speaking immigrants have not yet been investigated.

**Methods:** This single-arm pilot study (*N* = 30) explored the feasibility, acceptability, and cultural aspects of Mindfulness Training for Primary Care (MTPC)-Portuguese among Portuguese-speaking immigrants in the Boston area. MTPC is an 8-week, primary care-adapted, referral-based, insurance-reimbursable, trauma-informed MBI that is fully integrated into a healthcare system. The study also examined intervention preliminary effectiveness on mental health outcomes (depression and anxiety symptoms) and self-regulation (emotional regulation, mindfulness, self-compassion, interoceptive awareness), and initiation of health behavior was explored.

**Results:** Primary care providers referred 129 patients from 2018 to 2020. Main DSM-5 primary diagnoses were depression (76.3%) and anxiety disorders (6.7%). Participants (*N* = 30) attended a mean of 6.1 (SD 1.92) sessions and reported a mean of 213.7 (SD = 124.3) min of practice per week. All survey finishers would recommend the program to a friend, found the program helpful, and rated the overall program as “very good” or “excellent,” and 93% would participate again, with satisfaction mean scores between 4.6 and 5 (Likert scale 0–5). Participants and group leaders provided feedback to refine MTPC-Portuguese culturally responsiveness regarding materials language, settings, time, food, and community building. Patients exhibited reductions in depression (*d* = 0.67; *p* < 0.001) and anxiety (*d* = 0.48; *p* = 0.011) symptoms, as well as enhanced emotional regulation (*d* = 0.45; *p* = 0.009), and among survey finishers, 50% initiated health behavior change through action plan initiation.

**Conclusion:** This pilot study suggests that MTPC-Portuguese is feasible, acceptable, and culturally appropriate among Portuguese-speaking patients in the Boston area. Furthermore, the intervention might potentially decrease depression and anxiety symptoms, facilitate health behavior change, and improve emotional regulation. MTPC-Portuguese investigation with larger samples in controlled studies is warranted to support its dissemination and implementation in the healthcare system.

**Clinical Trial Registration:** Identifier: NCT04268355.

## Introduction

Immigrants in the United States (U.S.) are at a high risk of developing mental health disorders and experiencing mental healthcare inequalities and disparities (1–4). Portuguese is the seventh most widely spoken language in the world and over 540,000 foreign-born Portuguese-speaking individuals, sharing a language, a common history, and cultural traditions, live in the U.S. (5, 6). Massachusetts is the state with the largest Portuguese-speaking population, of whom 42% were born in Brazil, 28% in Portugal, 18% in Cape Verde, and 12% in the Azores (7). Boston is the top concentration area for Brazilian immigrants with 51,000 people (1.1% of the area population) (8).

Portuguese-speaking immigrants (PSI) experience high levels of psychological distress, depression, and anxiety (9–15). Unhealthy behaviors such as smoking and the lack of exercise and cancer screening are more prevalent when compared to other U.S residents (9). Socioeconomic disadvantages, language barriers, separation from family and friends, uninsurance and inadequate access to healthcare, discrimination, and fear of deportation are factors that contribute to health disparities (7, 11, 12, 16–18). Immigrants from Brazil work mostly with construction, house cleaning, and food services, being exposed to chemical, ergonomic, physical, and psychosocial job hazards (19). Research conducted with Brazilian immigrants in Massachusetts found that around one-third (35.3%) present significant depressive symptoms that are correlated with low income, lack of proficiency in English, being unmarried, and having a poor self-perception of health (10).

The literature demonstrates that mindfulness-based interventions (MBIs) are effective for improving physical and mental health outcomes, including depression and anxiety; reducing harmful health behaviors; and catalyzing chronic disease self-management and health behavior change (20–27). Meta-analytic data support the efficacy of MBIs to improve mental health and quality of life in primary care settings—the main gateway for patients in a healthcare system (28). As primary care settings are more accessible and less associated with social stigma around mental healthcare, it might be an ideal location for offering early intervention among immigrants (29). Furthermore, the group-based models make MBIs potentially more affordable and scalable for immigrants with financial constraints.

There is still a paucity of cultural and ethnoracial diversity among participants and group leaders in the MBI literature. The vast majority of MBIs have been studied in highly educated, English-speaking, and economically advantaged populations, leading to criticism of the low external validity of these interventions, especially in contexts of cultural and socioeconomic diversity (26, 30, 31). Developing a research base, while respecting cultural traditions from PSI, as well as other minorities such as African- and indigenous Americans, is needed (32, 33). To our knowledge, the feasibility and acceptability of MBIs among PSI in the U.S. have not yet been investigated.

*Mindfulness Training for Primary Care* (MTPC) is an 8-week, primary care-adapted, referral-based, insurance-reimbursable, trauma-informed, mindfulness-based intervention that is fully integrated into a healthcare system. This pilot study aimed to evaluate the feasibility and acceptability of a linguistic and cultural adaptation of MTPC for Portuguese speakers (MTPC-Portuguese). Additionally, we explored the preliminary effectiveness of the intervention on mental health outcomes (depression and anxiety symptoms), self-regulation (emotional regulation, mindfulness, self-compassion, interoceptive awareness), and the initiation of health behavior change among PSI.

## Materials and Methods

### Participants and Settings

We recruited adults between 18 and 70 years of age who received primary care within a participating primary care patient-centered medical home (PCMH) and who indicated Portuguese as their primary language in the electronic health record or as part of the referral to the MTPC program (recruitment process described elsewhere) (25, 34). All participants had a DSM-5 diagnosis of anxiety, depression, or stress-related disorder and had Portuguese fluency at sixth grade reading level. Exclusion criteria were the presence of symptoms of psychosis, thought disorder, and/or severe mental illness including schizophrenia, schizoaffective disorder, bipolar I disorder, current severe episode of major depressive disorder, active moderate–severe substance use disorder, cognitive impairment, high risk of imminent hospitalization (including current suicidal ideation or an inpatient admission or psychiatric emergency department visit in the last 6 months), third-trimester pregnancy, or an insurance payer that did not cover group medical visits.

**Figure 1:**
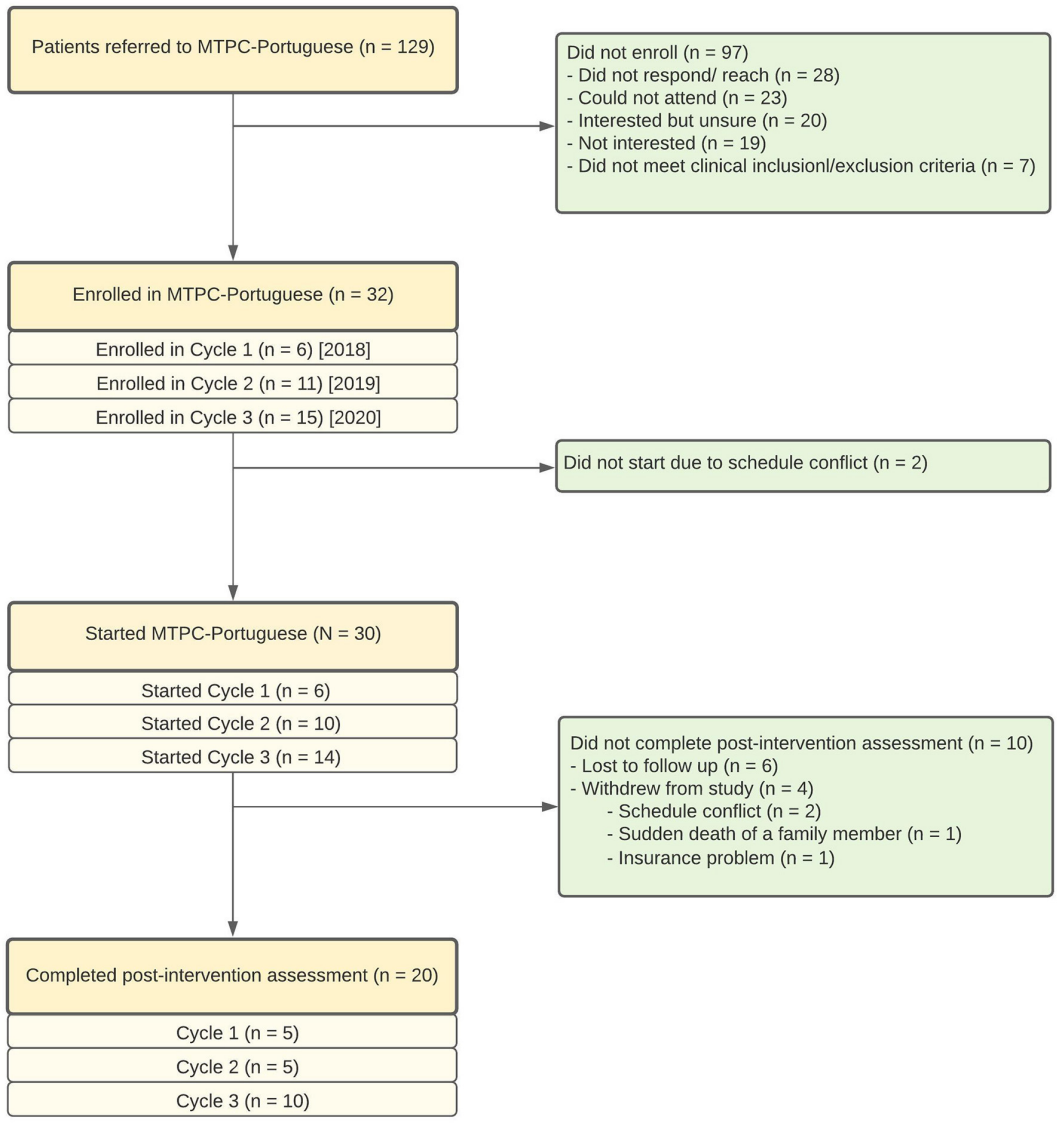
Flow diagram of the participants.

Mindfulness Training for Primary Care (MTPC) (25)-Portuguese was delivered in 8 weekly 2-h evening sessions over three cycles from 2018 to 2020 (Figure 1). Sessions were held from 6 to 8 p.m. in a community room in the building that housed one of the PCMH sites of the health system, as well as the Portuguese Mental Health Clinic. The 2020 recruitment cycle was disrupted by the COVID-19 nation-wide pandemic shutdown. The two evening groups and the all-day session of that 2020 cycle were offered interactively *via* an online *Google Meets* videoconference platform used by the healthcare system. While the primary physiologic aim for that 2020 recruitment cycle group (NCT04268355) was disrupted due to the inability to have in-person study visits, the main secondary clinical aims, mechanistic survey battery, and the intervention methods otherwise remained consistent across all three cycles, which represent the focus of this paper. All procedures performed were in accordance with the ethical standards of the institutional and/or national research committee and with the 1964 Helsinki Declaration and its later amendments or comparable ethical standards. Informed consent was obtained from all individual participants involved in the study which was approved by the CHA (Cambridge Health Alliance) Institutional Review Board (#1002/8/14).

### Procedures

A single-arm pre- and post-evaluation open trial was employed and measures were collected at baseline (week 0) and at post-intervention (weeks 8–9) using the online REDCap electronic data capture system (35). As part of the institutional change process (36, 37) resulting from integrating mindfulness within the health system (25), primary care providers (PCPs) and mental health providers were educated about this opportunity, the referral process and inclusion/exclusion criteria through emails, grand round presentations, and in-person presentations at all-staff meetings. Printed flyers were displayed in Portuguese and English at PCMHs informing potential patients to contact their PCP or mental health provider about eligibility, as well as a reminder was sent to PCPs and mental health teams. Interested primary care patients were referred by their PCP or mental health provider *via* a customized referral order in the electronic health record in which primary and secondary referral diagnoses were indicated. A convenience sample was used, and providers were encouraged to refer patients who had comorbid mental and physical conditions who had an interest in mindfulness and mind–body approaches to managing their chronic illness, or who were interested in a group-based approach to managing depression, anxiety, or stress. All referrals underwent a preliminary chart review by a study coordinator. A board-certified psychiatrist reviewed diagnosis and eligibility if the preliminary chart review was unclear (TG). To assess clinical appropriateness and confirm diagnosis for insurance billing, patients were required to have a behavioral health evaluation with a CHA provider within the past 6 months and may have received additional mental health treatment based on the stepped care model of the system (38). Behavioral health evaluations were held in Portuguese when the mental health provider spoke Portuguese or with the support of a CHA English-Portuguese medical interpreter when not.

Eligible individuals were invited to an orientation group session coordinated by members of the research staff that spoke Portuguese (MT, LC, AO, AR, NM). During the session, detailed information about the study and time to ask questions were provided, culturally accepted food was offered, informed consent was signed, and baseline measures were collected. This session also included a conceptual/experiential mindfulness introduction and inquiry by the Portuguese-MTPC facilitator (AO, MT), and digital (39)/community mindfulness resources in Portuguese and English were shared to enhance patient motivation and practice opportunities. During the intervention, participants received a biweekly engagement call to provide study staff support (i.e., logistical, emotional, mindfulness-practice encouragement) and reduce attrition. Recruitment and engagement calls were conducted by research staff who were Portuguese speakers (LC, AR, NM, MT). Sessions were audio-recorded, and 10% were reviewed by trained observers for adherence and competency, preventing drift. All Portuguese-MTPC groups were billed as group medical visits.

### Translation of Materials

MTPC-Portuguese is a first phase primarily linguistic MTPC cultural adaptation developed in order to provide MTPC access to PSI. MTPC materials were translated by two Portuguese native speakers and mindfulness experts fluent in English (MH, MD) together with a primary care physician and experienced mindfulness facilitator who co-developed the MTPC curriculum and was previously trained and worked as an English-Portuguese medical interpreter (AO). Research materials were translated by research staff Portuguese native speakers (MH, MT, LC, AR) in combination with CHA translation services and revised by at least one other Portuguese native speaker from the research staff.

### Group Leaders

Cycles 1 and 2 were led by a primary care physician and mindfulness group leader who co-developed the MTPC curriculum, had lived in Brazil, and was previously trained and worked as an English-Portuguese medical interpreter (AO), leading groups since 2012. Cycle 3 was co-led in pairs by the aforementioned primary care physician with a psychiatrist and mindfulness group leader (MT) who is a Portuguese native speaker leading groups since 2015. Both group leaders were qualified in the Center for Mindfulness and Compassion at CHA/Harvard Medical School, led over 25 mindfulness groups, and regularly lead at least one new group per semester and weekly 45–60 min mindfulness maintenance sessions.

### Intervention

MTPC builds upon the transdiagnostic approach developed in mindfulness-based stress reduction (MBSR) (40) and combines training in evidence-based targeted mindfulness skills from other MBPs (41–44) with elements from mindfulness-based cognitive therapy (MBCT) (44) and approaches to behavior change adapted from cognitive behavioral therapy (45), relapse prevention (46), and motivational enhancement (47). MTPC is typically offered in 8 weekly 2-h sessions with a 7-h all-day optional session and a recommended 30–45 min of daily home practice with online recordings. MTPC was designed to be trauma-informed (48, 49) through, for example, availability of choice in guided practices; language during meditations that emphasizes freedom, choice, self-compassion, and self-care; and explicit modules during MTPC group leader training on trauma adaptations and the ubiquity of traumatic experiences. Foundational sessions 1–4 fostered awareness of body sensations, breathing, autopilot and stress responses, and skills for relating to discomfort. Sessions 5–8 included MTPC-incorporated core practices adapted from MBCT (44) and mindful self-compassion (MSC) (43). The STOP-ACHE-GO practice (see Supplementary Material) invites participants to bring awareness to processes that are gradually introduced over the program with a new letter of the acronym. A thread focused on “Living Well” with chronic illness was woven throughout most sessions and included harnessing mindfulness and chronic illness self-management (50) to support health behavior change as a way of living well.

Since ambivalence is often rooted in a conflict of values, and behavior change emerges from becoming aware of the discrepancy between deeply held values and current behavior (51), MTPC avoids promoting any specific values, but uses mindfulness practice to provide a safe and illuminating container for the identification of one's own deeply held values through an adapted values clarification card sort process (52), followed by encouragement to identify which important personal values are associated with living well. Finally, MTPC includes a short-term action planning process aimed at behavior change related to health maintenance or chronic illness self-management using the SMART model (53). Table 1 describes the MTPC curriculum themes, goals, and activities.

**Table 1:** Mindfulness training for primary care (MTPC) core curriculum content.

Sessions*	**Goals**	**Practices/activities**	**Homework**
1. Discovering mindfulness and autopilot	Developing mindfulness and noticing health behaviors (sessions 1–4)	Intention setting Standing mindful movement Raising exercise Body scan	Body scan Tracking autopilot in 10 ways STOP-ACHE-GO (S-A-G)
2. Perception, interpretation, and beginner's mind		Body scan Friend walking down the street (automatic thinking/emotions exercise, negativity/threat bias) Curiosity about health habits—journal Awareness of sounds, posture, and breathing	Body scan Notice autopilot triggering cues S-A-G
3. Finding freedom through feeling tone		Sitting body scan with feeling tones Mindful movement Understanding the feeling tone by observing the diamond of experience Mindful savoring	Mindful movements Body scan Diamond of experience for pleasant and unpleasant events Mindful savoring
4. Staying present with pain and stress; allowing what is		Sitting with discomfort Understanding our experience of stress on the body Mindful walking 3-min S-A-G breathing space	Sitting meditation Mindful walking, 3-min S-A-G breathing space Noticing warning signs S-A-G
5. Kindness and coping	Compassion and preparing for behavior change (sessions 5–8)	Kindness meditation 3-min S-A-G self-compassion break Journal about health challenge Giving and receiving compassion meditation	Sitting meditation Kindness meditation S-A-G How can I best take care of myself?
6. Accessing core values, aspiration, and change		Sitting meditation Using mindfulness to access core values S-A-G breathing space with grounding values and gratitude	Sitting meditation, kindness meditation giving, and receiving compassion meditation S-A-G Gratitude journal
7. Living well through wise action		SMART action plan video/ behavior change action plan creation Urge surfing with S-A-G Sitting meditation	Sitting meditation, kindness meditation giving, and receiving compassion meditation S-A-G Start behavior change action plan
8. Connection, communication, and community		Kind mind meditation Noticing interdependence/Who has a stake in your well-being Community resources	Incorporate mindfulness into daily life

* *Every session incorporates the following 3 threads, which develop and deepen over the 8 weeks. Thread 1: the role of behavior change in living well. Thread 2: development of interpersonal mindfulness. Thread 3: warmth, kindness, compassion, and the experience of common humanity. A 7-hour silent all-day optional session is offered on a weekend between weeks 5–8*.

### Measures

Participants completed a baseline survey for sociodemographic variables, including gender, age, country of origin, race, income, marital status, and education.

#### Feasibility, Acceptability, and Cultural Aspects

The recruitment process, MTPC-Portuguese sessions attendance, and dropout rate were evaluated. Participants were invited to record daily practice/resource variables weekly until week 8 on a REDCap link, including formal practice (e.g., body scan), informal practice (i.e., breathing space, mindful walking, mindful eating, body awareness, gratitude, informal kindness, self-compassion break), and use of mindfulness resources (e.g., online recordings).

The *MTPC-Portuguese Satisfaction Survey* (available as Supplementary Material) is an 18-item survey containing two parts. First is a series of 12 questions scored on a five-point Likert scale from 1 (*Strongly Disagree/Poor*) to 5 (*Strongly Agree/Excellent*), with statements such as “*I would recommend this program to a friend*,” “*I found this program helpful*,” “*I would be willing to participate in this program again*,” or “*Overall rating of the program*.” Next is a series of six open-ended questions in which patients enter a written response to statements such as “*The most important thing I learned during this program*” or “*My favorite part of the program*.” The MTPC-Portuguese Suggestion Survey containing the four following questions was included in cycle 3 requesting suggestions to improve cultural responsiveness in case of negative answers. (1) “*Was the language presented in the didactic or audiovisual content material not clear enough or inappropriate to your cultural perspective? Consider metaphors, vignettes, poems, sayings and symbols utilized*” (cognitive); (2) “*In any proposed activity, was there any message communicated that does not fit your norms, values, or cultural traditions that may have created resistance during the program?*” (affective); (3)“*Are the program structure and content delivery in the sessions, as well as the formal and informal home practices are applicable to your daily life experience?*” (relevance); (4) “*In order to make the program more accessible to your culture, do you have any other suggestions for changes not covered in the previous questions?*” A semistructured 35-min interview with group leaders following the Suggestion Survey was conducted and recorded by a research staff member (LC) and discussed with the research team (MD, LC, RG, NR, ZSO) in order to assess the perspectives of leaders on cultural aspects.

#### Mental Health

The *Patient-Reported Outcomes Measurement Information System—Depression Short Form 8a (PROMIS-DSF)*, an eight-item scale, was used to assess patient-reported health status for depression (54). PROMIS instruments are funded by the NIH and used to reliably and validly measure patient-reported outcomes for clinical research and practice. Participants were asked to rate their experience of each item in the past 7 days on a five-point scale from 1 (*never*) to 5 (*always*) (Cronbach's α = 0.94). The PROMIS-Depression Portuguese version demonstrates strong psychometric properties (Cronbach's α = 0.97) (55).

The *Patient-Reported Outcomes Measurement Information System—Anxiety Short Form 8a (PROMIS-ASF)*, an eight-item scale, was used to assess patient-reported health status for anxiety (56). PROMIS instruments are funded by the NIH and used to reliably and validly measure patient-reported outcomes for clinical research and practice. Participants were asked to rate their experience of each item in the past 7 days on a five-point scale from 1 (*never*) to 5 (*always*) (Cronbach's α = 0.90). The PROMIS-Anxiety Portuguese version exhibits strong psychometric properties (Cronbach's α = 0.96) (55).

#### Self-Regulation

The *Difficulties in Emotion Regulation Scale (DERS)* is a 36-item self-report scale used to assess emotional dysregulation using a five-point Likert scale ranging from 1 (*almost never*) to 5 (*almost always*) (57). The scale assesses six aspects of emotional dysregulation: non-acceptance of emotional responses (Non-acceptance), difficulties engaging in goal-directed behavior (Goals), impulse control difficulties (Impulse), lack of emotional awareness (Awareness), limited access to emotion regulation strategies (Strategies), and lack of emotional clarity (Clarity). Subscales are summed and a lower total score represents a better outcome (α = 0.93). The DERS Portuguese version demonstrates adequate psychometric properties (α = 0.93) (58).

The Five *Facet Mindfulness Questionnaire (FFMQ)* is a 39-item scale used to examine five factors that represent aspects of the current empirical conception of mindfulness (59). Participants rated their degree of agreement with each of the items on a five-point Likert scale ranging from 1 (*never or very rarely true*) to 5 (*very often or always true*), with higher scores indicating higher experience of mindfulness (α = 0.93). The FFMQ Portuguese version shows good psychometric properties (α = 0.81) (60).

The *Self-Compassion Scale (SCS)* is a 26-item scale used to measure six components of self-compassion: self-kindness, self-judgment, common humanity, isolation, mindfulness, and overidentification (61). The items are rated on a five-point response Likert scale ranging from 1 (*almost never*) to 5 (*almost always*) (α = 0.93). The Portuguese SCS version exhibits solid psychometric properties (α = 0.92) (62).

The *Multidimensional Assessment of Interoceptive Awareness (MAIA)* is a 32-item self-report scale used to assess multiple aspects of interoception and interoceptive awareness (63). The six-point Likert scale (ranging from 0 to 6) assesses eight aspects of interoceptive awareness: noticing, not-distracting, not-worrying, attention regulation, emotional awareness, self-regulation, body listening, and trusting. Subscales are averaged, and a higher total score represents a better outcome (α = 0.66 to 0.87). The MAIA Portuguese version presents good psychometric properties (α = 0.61 to 0.87) (64).

#### Health Behavior Change

During study week 7, participants created a short-term action plan focused on behavior change related to health maintenance and/or self-management of chronic disease using video and written materials outlining the well-established SMART goal framework (53, 65). Participants then reported their level of action plan initiation in the *Action plan initiation (API) survey* (66), from 1 (*not at all*) to 7 (*completely*) at week 9, a 2-week time window consistent with previously published studies (66–68). Evidence of action plan initiation was defined as an API score ≥5.

#### Adverse Events

Adverse events reports (AERs) were collected using a combination of checklist and open-ended questions in the intervention period during biweekly engagement calls in Portuguese (LC, AR, NM) and at postintervention. Research staff documented any AERs occurring during group sessions. AERs were categorized as serious or non-serious. Serious adverse events were previously defined as any adverse event that resulted in one or more of the following outcomes: death, life-threatening event, inpatient hospitalization, a congenital anomaly or birth defect, or an important medical event based upon appropriate medical judgment. AERs were classified according to the likelihood that they were related to the intervention using a Relatedness Assessment Tool.

### Data Analysis

Descriptive statistics were used to evaluate baseline demographics and clinical characteristics of participants, feasibility, and acceptability. Qualitative feedback was also used to explore acceptability and cultural aspects.

To examine mental health and self-regulation variables, we conducted a repeated measures analysis using linear mixed-effects models with a fixed time parameter for 8 weeks vs. baseline and participant-specific random intercepts. Given that the study design included observation of continuous outcome measures for each participant at two time points, linear mixed models were an appropriate choice because they account for clustering (i.e., non-independence) of multiple observations per participant (69). Within-group effect sizes were calculated and expressed in terms of Cohen's *d*. We used multiple imputation by chained equations with predictive mean matching and 100 imputations to address missing outcome variable data, which ranged from 3 to 33% across all measures (70, 71). Statistical significance of pre-/post-differences was determined using the Benjamini–Hochberg false discovery rate (FDR) procedure (72), which accounts for multiple comparisons. We implemented the FDR procedure according to Cao et al. (73) in which a cutoff *p*-value is determined for a family of similar variables and analyses (family-wise error rate = 0.05) (74, 75). We designed two analysis families: mental health outcomes (depression and anxiety symptoms; two items) and self-regulation outcomes (four total scale items, six emotion regulation-specific subscale items, and eight interoceptive awareness-specific items). Independent *t*-tests and Pearson's correlations were conducted to determine if there were any differences in baseline mental health, self-regulation, and demographic variables between patients who did and did not answer the postintervention assessment. We added gender as a covariate to each mixed-effects model to test whether there were significant, independent differences in mental health and self-regulation outcomes between females and males after controlling for time.

To prevent bias during analysis, an external statistical consultant (TC) oversaw the analysis plan and decision-making and reviewed all Stata/MP 16.1 (76) results and syntax.

## Results

Participants (*N* = 30) were 80% female (*n* = 24) and had a median age of 52 years old, and 30% identified themselves as Black or mixed race (*n* = 9). Participants were 93.3% (*n* = 28) immigrants to the U.S. born in Brazil, while 6.7% (*n* = 2) were immigrants born in Portugal. There were no participants from other Portuguese-speaking countries. Annual income below US$20,000 was reported by 43.3% (*n* = 13) of the sample. Main DSM-5 primary diagnoses were depression (76.3%, *n* = 23) and anxiety disorders (6.7%, *n* = 2), whereas 16.7% (*n* = 5) of participants suffered from comorbid depression and anxiety disorders. Baseline demographic and clinical characteristics are described in Table 2.

**Table 2:** Baseline demographic and clinical characteristics of participants.

**Variable**	**Total** **(** ***N*** **=** **30)**	
Female, *n* (%)	24	(80.0)
Age (years), median (IQR)	52	(45-61)
**Country of origin**, ***n*****(%)**		
Brazil	28	(93.3)
Portugal	2	(6.7)
**Race**, ***n*****(%)**		
White	29	(63.3)
Black	3	(10.0)
Mixed	6	(20.0)
Missing	2	(6.7)
Annual income < $20,000, *n* (%)	13	(43.3)
Missing	1	(3.3)
**Marital status**, ***n*****(%)**		
Single	9	(30.0)
Married/cohabitating	12	(40.0)
Divorced	7	(23.3)
Widowed	1	(3.3)
Missing	1	(3.3)
Education (years), median (IQR)^a^	14	(14-16)
**Insurance type**, ***n*****(%)**		
Medicare/Medicaid	28	(93.3)
Private	1	(3.3)
Other^b^	1	(3.3)
Single DSM-5 diagnosis, *n* (%)	22	(73.3)
**Primary DSM-5 diagnosis**, ***n*****(%)**		
Major depressive disorder^c^	21	(70.0)
Unspecified anxiety disorder (309.1)	2	(6.6)
Other depressive disorder^d^	2	(6.3)
Adjustment disorder^e^	1	(3.3)
PTSD (309.81)	1	(3.3)
Other^f^	3	(10.0)

*^a^Missing (n = 1).*

*^b^Compensation of workers (n = 1).*

*^c^Includes DSM-5 codes: major depressive disorder, single episode, unspecified (296.20); major depressive disorder, single episode, moderate (296.22); major depressive disorder, recurrent episode, unspecified (296.3); major depressive disorder, recurrent episode, mild (296.31); major depressive disorder, recurrent episode, moderate (296.32); major depressive disorder, recurrent episode, severe (296.33); major depressive disorder, recurrent episode, in partial remission (296.35); major depressive disorder, recurrent episode, in full remission (296.36).*

*^d^Includes DSM-5 codes: dysthymia (300.4) and unspecified depression (311).*

*^e^DSM-5 codes: adjustment disorder, with anxiety (309.24).*

*^f^Includes DSM-5 codes: somatic symptom disorder (300.82); acute stress disorder/reaction (308.3)*.

### Feasibility

Over three cycles, 129 patients were referred and 32 signed informed consent (Figure 1). The main reasons for not enrolling were that people were unable to be reached or could not attend. Participants who initiated MTPC-Portuguese (*N* = 30) attended a mean of 6.1 (SD 1.92) sessions, 86.7% (*n* = 26) attended at least four sessions, and 70% (*n* = 21) attended at least 6 of the 8 weekly sessions (Table 3). Average formal practice reported by participants was 213.7 minutes (min)/week (SD = 124.3) or 30.5 min/day. Body awareness (2.46 counts/week), mindful eating (2.37 counts/week), and breathing space (1.59 counts/week) were the predominant reported informal practices, whereas MTPC online recordings, other center/facilitator online recordings, and mindfulness books/articles were the most frequent resources used, with an average of 3.09, 1.29, and 1.1 counts per week, respectively.

**Table 3:** Number of sessions completed by participants (*n* = 30).

**Number of sessions**	***N* (%)**
1	30 (100.0)
2	29 (96.7)
3	28 (93.3)
4	26 (86.7)
5	23 (76.7)
6	21 (70.0)
7	16 (53.3)
8	9 (30.0)

Post-intervention assessment was completed by 62.5% (*n* = 20) of the enrolled sample. To assess bias due to attrition, independent *t*-tests were conducted to compare participants that did and did not complete (*n* = 10) the postintervention assessment on baseline depression, anxiety, emotion regulation, mindfulness, self-compassion, and interoceptive awareness and found no differences (*p* > 0.05). Additionally, independent *t*-tests and Pearson's correlations revealed that gender, age, country of origin, race, income, marital status, and years of education were also similar between the groups (*p* > 0.05).

### Acceptability

Satisfaction survey results demonstrated that 100% of responders would recommend the program to a friend, 100% found the program helpful, 93% would participate again, and 100% rated the overall program as “very good” or “excellent (4 or 5 on the Likert scale). Satisfaction survey mean scores ranged from 4.6 to 5 and are shown in Table 4.

**Table 4:** Satisfaction scores of MTPC-Portuguese participants (*n* = 15).

**Question**	**Mean scores (1-5)**
1. I found this program helpful	4.9
2. The group was well-organized	4.9
3, The group leader(s) care about me as a person	5.0
4. I was able to participate and express myself in the group	4.8
5. The group leader(s) were authentic, honest, and real	5.0
6. I learned what I was hoping to learn	4.6
7. The group leader(s) had good timing when providing examples	5.0
8. I would be willing to participate again if I was able to do so	4.9
9. The group leader(s) were easy to understand	5.0
10. I would recommend this program to a friend	5.0
11. Overall rating of the facilitator(s)	5.0
12. Overall rating of the group	4.9

Mindfulness skills and attitudes developed through the program had a positive impact on the daily life of participants. “*I learned to pay attention to present-moment feelings*” (female, 37 years old), “*Live more consciously*” (female, 30 years old), “*Cultivate non-judgement*” (female, 54 years old), “*Concentration*” (female, 62 years old), and “*Step out of the automatic pilot*” (male, 63 years old) were the responses to the most important or favorite part of the intervention questions.

A central theme highlighted in the qualitative feedback was the importance of social connectedness and feelings of common humanity provided by the group expressed in answers regarding the favorite or most helpful part of the program such as “*Meeting new people and listening to their experiences*” (female, 42 years old) and “*I realized I am not alone*”; “*making new friends*” (female, 37 years old). Another prominent aspect was the cultivation of inner compassion and acceptance, highlighted in answers to the most important part of the program like “*I learned that I have the capacity to love and forgive myself, and live well with my limitations*” (male, 61 years old) or “*The practice of being kind, caring and accepting toward myself* ” (male, 53 years old). “*I learned to stay calm in difficult situations*” (female, 37 years old), “*Deal with anxiety*” (female, 35 years old), and “*Self-control*” (female, 62 years old) were the answers to the most helpful aspect of MTPC-Portuguese, indicating that new skills to deal with challenging emotions and impulsivity were fostered.

The importance of formal mindfulness and compassion/loving-kindness practices and the all-day session was demonstrated in responses to the favorite or most important part of the intervention such as “*All-day session*” (female, 54 years old), “*Body scan*” (female, 68 years old), and “*Mindful movement and compassion practices*” (female, 30 years old). Feedback received regarding the least favorite part of the program or suggestions for change centered around program length and settings like “*The program could be longer*” (female, 37 years old), “*Add 15 min per session*” (female, 30 years old), or “*An alternative space without the Health Unit public address system interruptions*” (male, 53 years old).

### Cultural Aspects

Regarding cultural aspects, 100% of survey completers considered the formal and informal home practices applicable to daily life. When asked if there was any message communicated that did not fit participant norms, values, or cultural traditions that could have created resistance or if the intervention structure and content delivery in the sessions, all survey completers answered no. Portuguese-MTPC language in the didactic or audiovisual content material was overall clear and appropriate, though one participant mentioned the program workbook would benefit from a review of poetry translations. One participant stated that the program content welcomes all faiths and suggested making it clearer in the recruitment process because a few of her friends who could have benefited were advised by religious leaders (Christian) not to participate.

Both group leaders reported that the Portuguese-MTPC appropriately considered cognitive, affective, and relevance aspects. However, the group leaders had a few suggestions to improve cultural responsiveness such as providing additional time, space, and resources to meet social connection necessities; including poetry and testimonials of Portuguese-speaking authors; offering traditional food from Portuguese-speaking countries; highlighting the spiritual/religious inclusiveness of the intervention (especially its compatibility with Christian beliefs); and inviting participants to watch a video at the first session that filmed a previous Portuguese-speaking MTPC group participant sharing his or her experience with the program.

### Mental Health

**Figure 2:**
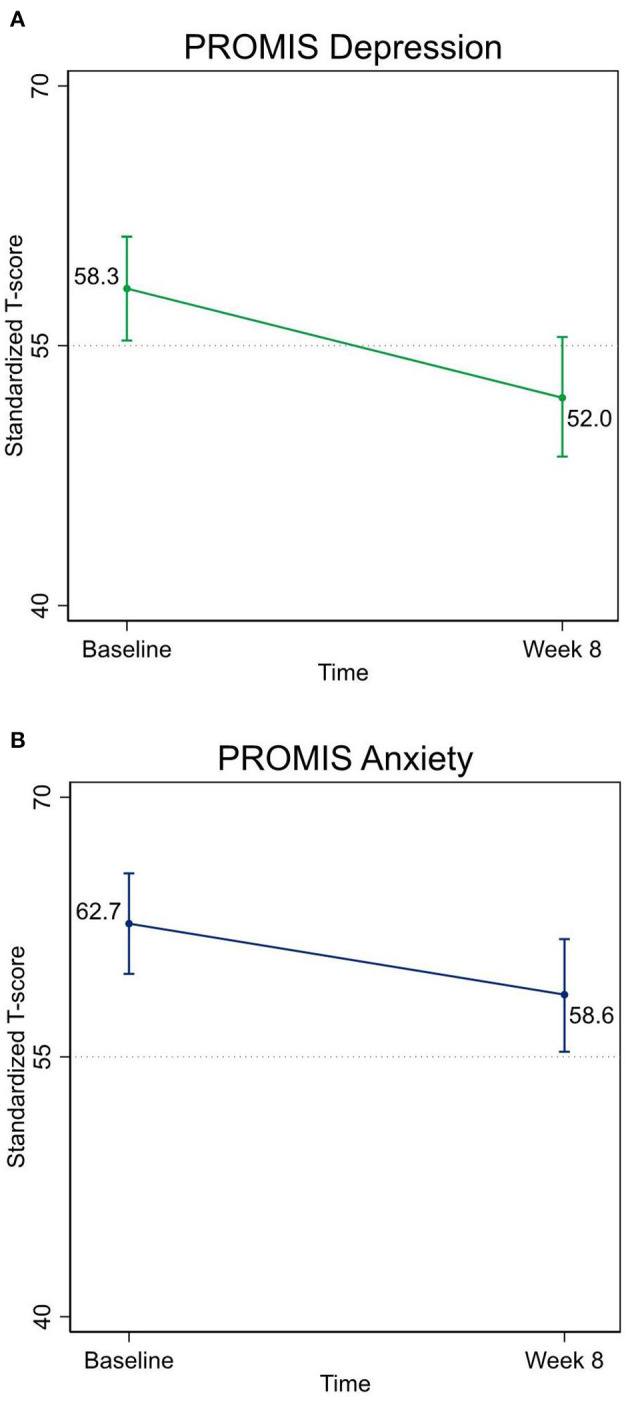
**(A)** Pre-/post-reduction in depression symptoms. **(B)** Pre-/post-reduction in anxiety symptoms. PROMIS Depression, Patient-Reported Outcomes Measurement Information System—Depression Short Form 8a; PROMIS Anxiety, Patient-Reported Outcomes Measurement Information System—Anxiety Short Form 8a.

Mixed models analysis demonstrated that MTPC-Portuguese was associated with a medium-to-large effect size in reducing depression symptoms (*d* = 0.67; *p* <0.001) and a medium effect size in decreasing anxiety symptoms (*d* = 0.48; *p* = 0.011) (Table 5 and Figure 2). The percentage of patients that scored above 55 on PROMIS-DSF and PROMIS-ASF (the cutoff strongly associated with disorder) (77, 78) at baseline was 70 and 83%, respectively. Sensitivity analyses for the mental health outcomes on this sample were conducted, and significant findings were found that were similar to the overall sample analysis. Gender was not yielded as a significant covariate for the mental health outcomes.

**Table 5:** Mixed-effects analysis.

**Outcome**	**Baseline**		**Week 8**		**Differences over time**			
	**Mean**	**(SE)**	**Mean**	**(SE)**	***B***	**(SE)**	***p***	***d***
PROMIS-DSF^a^	58.2	(1.5)	52.1	(1.8)	−6.2	(1.6)	<0.001*	0.67
PROMIS-ASF^b^	62.7	(1.5)	58.5	(1.7)	−4.1	(1.6)	0.011*	0.48
DERS^c^ total	91.7	(4.4)	81.0	(5.0)	−11.3	(4.3)	0.009*	0.45
FFMQ^d^ average	3.1	(0.1)	3.3	(0.1)	0.2	(0.1)	0.115	0.35
SCS^e^ average	3.2	(0.1)	3.5	(0.2)	0.3	(0.2)	0.188	0.31
MAIA^f^ average	3.5	(0.1)	3.8	(0.1)	0.3	(0.2)	0.039^∧^	0.45

*^a^Patient-Reported Outcome Measurement Information System—Depression Short Form 8a.*

*^b^Patient-Reported Outcome Measurement Information System—Anxiety Short Form 8a.*

*^c^Difficulties in Emotion Regulation Scale.*

*^d^Five Facet Mindfulness Questionnaire.*

*^e^Self-Compassion Scale.*

*^f^Multidimensional Assessment of Interoceptive Awareness.*

^*^Significant after the Hochberg FDR procedure, family-wise p < 0.05.

*^∧^Significant before the Hochberg FDR procedure*.

### Self-Regulation

The MTPC-Portuguese intervention was also associated with a significant, small-to-medium effect size improvement in emotion regulation (total DERS score, Table 5: *d* = 0.45; *p* = 0.009). Among the DERS subscales, significant decreases in the *Non-acceptance* (*d* = 0.50; *p* = 0.013) and *Strategies* (*d* = 0.54; *p* = 0.004) items appeared to drive the improvement in overall emotion regulation (see Supplementary Material). Though not statistically significant, there were trends toward increased mindfulness (*d* = 0.35; *p* = 0.115), self-compassion *d* = 0.31; *p* = 0.188), and interoceptive awareness (*d* = 0.45; *p* = 0.039; not significant after controlling for multiple comparisons through the false discovery rate) (Table 5). None of the estimates for changes in the eight individual MAIA scales were statistically significant. Gender was not yielded as a significant covariate for FFMQ, SCS, DERS, and MAIA total scores.

### Health Behavior Change

Among survey finishers, 50% initiated the action plan by week 9 (API score ≥ 5). Action plans were individualized (e.g., “practice the body scan for 15 min 3 times a week,” “run 3 times a week,” or “go to the gym 2 times a week”). The most prevalent action plan goal category was mindfulness or self-care (58%), followed by physical exercise (17%), diet (17%), and other aspects impacting health (8%).

### Adverse Events

No serious adverse events and two non-serious adverse events were reported during the study. Adverse events were unlikely related to the intervention. One participant experienced knee pain related to osteoarthritis, consulted with CHA orthopedics team, and continued in the study. Symptoms improved over the weeks of the study. Another participant experienced the sudden death of a family member and reported worsening of anxiety and depression symptoms. After consultation with the group leader, patient mental health provider, and research staff, the participant was withdrawn from the study and received specialized treatment from the CHA mental health team.

## Discussion

The findings from this pilot study suggest that MTPC-Portuguese is feasible, acceptable, and culturally appropriate among low-income PSI in primary care settings. Low dropout and a high attendance rate comparable to MTPC studies in English (25, 34), standard MBIs (79), and superior to other community-based MBSR studies with immigrants from Ibero-American countries (80–82) indicate the potential feasibility of the intervention and should be underlined, since low-income immigrants commonly experience attendance obstacles such as transportation cost, long working-hours, and family obligations (19, 80, 83, 84).

The high attendance rate may be attributed to a trauma-informed intervention curriculum (25) that includes a significant amount of interpersonal mindfulness and mindfulness of the body practices (i.e., body scan, mindful movement), which were well-received and easily related to cultural expectations from PSI (85). In addition, the biweekly engagement calls, location within a primary care building, and the program integration with the health system, primary care, and mental health providers likely increased acceptability and attendance. The systematic review and meta-analysis of Parsons et al. on MBSR and MBCT formal practice over 8 weeks revealed an average of 29 min/day (86), which is similar to our findings. The fact that body awareness was the leading informal practice reported also illustrates the role of the body in the sample culture and its emphasis on MTPC curriculum content (25, 85). The use of mindfulness resources offered in Portuguese to participants, particularly online recordings and books/articles, could also have contributed to program overall engagement.

Potential feasibility and acceptability were strongly endorsed by elevated satisfaction scores and by responses which underscored program curriculum that combines traditional “cool” mindfulness with “warm mindfulness” —infused with self-compassion and inner warmth—emotional regulation strategies (26, 87, 88). The cultivation of open and accepting awareness, self-compassion, and self-care skills, together with the experience of shared common humanity, social connectedness, and support from peers and group leaders, also corroborated by the high satisfaction scores on questions 3, 4, and 5 [ “*The group leader(s) care about me as a person*,” “*I was able to participate and express myself in the group*,” and “*The group leader(s) were authentic, honest, and real*”] reported by participants, fulfills important needs of immigrants who often experience unresolved trauma, isolation, loneliness, shame, and disempowered status (11, 18, 89–91).

Valuable feedback that might be considered for MTPC-Portuguese culturally responsiveness refinement aiming to foster social connectedness, community building, and a sense of belonging and safety to share vulnerabilities are as follows: offering the program in a Portuguese-speaking community center, offering maintenance mindfulness sessions, sharing community activities regularly, providing space and time before/after the weekly session for participants to meet, providing traditional food, inviting a previous Portuguese-speaking MTPC participant to narrate his/her experience, reviewing English poetry translations, providing a supplement such as poetry from Portuguese-speaking authors, clarifying myths about mindfulness, and disclosing MTPC psychosocial science-informed features that welcome all spiritual beliefs. Even though sessions were held in the evening, many individuals reported not being able to enroll in the program due to the long weekday working schedule; therefore, offering the intervention on weekends or later in the evening could improve its accessibility. The feedback provided resonates with the culturally responsive literature on MBIs which highlights the importance of a safe and accessible space (33, 92); community-based partnerships, advisors, and resources (33, 92–96); supplementing reading material with culturally appropriate writings (93); and use of cultural-familiar and inclusive terminology avoiding terms like “meditation” (32, 33, 92, 93).

MTPC-Portuguese demonstrated a medium-to-large effect size in decreasing depression symptoms and a medium effect size in reducing anxiety symptoms, in accordance with a previous randomized controlled trial which showed that MTPC in English decreases anxiety when compared with a mindfulness low-dose comparator with significant within-group effect sizes ranging from *d* = 0.43 (depression) to *d* = 0.72 (anxiety) (34). The potential mental health benefits described herein are aligned with a small but growing literature described by Cotter et al. (97) in a recent review of MBI research among U.S. immigrants with origins in countries from Ibero-America, largely represented by uncontrolled trials, where five of six studies found a significant reduction in depressive symptoms (82, 98–101) and five of eight studies reported a significant impact on anxiety symptomatology (80, 82, 99, 100, 102) after mindfulness training. Research conducted in Brazil also suggests salutary effects of mindfulness training on depression and anxiety (103–105), though investigation in primary care settings is needed (106, 107). Though patients with a current severe episode of major depression were excluded, the extensive prevalence of major depression disorder combined with substantial symptomatology revealed by the percentage of individuals presented with at least mild depression and anxiety (scores >55 on PROMIS-DSF and PROMIS-ASF) denotes the sample mental and emotional suffering severity and may have contributed to the magnitude of the findings.

Since lower socioeconomic status and the acculturation process among immigrants are associated with higher morbidity, mortality, and harmful health behaviors (1–3, 9, 108–110), the finding that a significant proportion of patients initiated the health behavior action plan at rates similar to MTPC studies in English (25, 34) cannot be overemphasized. Emotional regulation, mindfulness, self-compassion, and interoceptive awareness appear to be synergistic self-regulation mechanisms through which MBIs exert their salutary effects (26, 111), at least among English speakers. Emotional regulation improvement points in the same direction as previous MTPC research (25). DERS subscales of *Non-acceptance* [i.e., non-acceptance of emotional responses (“When I'm upset, I become angry at myself for feeling that way”)] and *Strategies* [i.e., limited access to emotion regulation strategies (e.g., “When I'm upset, I believe there is nothing I can do to feel better”)] (112) drove the enhancement in this study. Interestingly, two studies conducted in the U.S. found that MBSR increased *Strategies* and *Goals* subscales (113, 114). Our findings may offer insights on which specific emotional regulation abilities are impacted by MBIs across cultures. The trend toward mindfulness, self-compassion, and interoceptive awareness improvement, yet with a lack of statistical significance, could be a result of our small sample size and type II error, or cultural differences in which mechanisms are most active, which deserves further research.

The empirical results reported herein should be considered in the light of some limitations and the interpretations conditioned to the exploratory nature of pilot studies. The sample was composed predominantly by female patients and does not fully represent the demographics of PSI. Brazilians were overrepresented, Portuguese were underrepresented, and immigrants from the Azores and Cape Verde were not represented, limiting the generalizability of the findings (7). The last two sessions of cycle 3 intervention were held online, and the all-day session was abbreviated (7 to 4 h) and online to protect the safety of patients when the COVID-19 pandemic started. Without a control group, causality cannot be inferred and results could be attributed to other time-related variable, including the fact that some participants received additional treatment through the stepped care model of the CHA with ongoing psychopharmacology or psychotherapy (76.6 and 30% of the sample that started intervention, respectively). Effect sizes should be interpreted with caution due to the small sample size. Finally, follow-up data were not collected to illuminate daily life incorporation of mindfulness practices and benefits over time. Despite these limitations, the study has several strengths including the following: *originality*—it was the first study we are aware of that evaluated MBI feasibility, acceptability, cultural aspects, and effects on PSI; *relevance*—it meets an important need for understanding how MBIs can be better suited to a vulnerable growing minority group, and also contributes to the evidence base for the feasibility of MBIs on immigrants and low socioeconomic status populations (26, 30); and *clinical implications for public health*—it was conducted in a real-life primary care clinical context which is the most common service domain where immigrants seek healthcare (115).

In summary, the results of this pilot study suggest that an 8-week primary care and linguistically adapted MBI is potentially feasible, acceptable, and culturally appropriate for PSI. Pilot studies are necessarily the first steps in exploring interventions, and further investigation and implementation of culturally relevant adaptations to underserved minorities is pivotal to enhance engagement and mitigate health disparities in the country. This continuous process should be approached with a beginner's mind and in close collaboration with culturally appropriate community members (33, 106–109). Additionally, MTPC-Portuguese might potentially decrease depression and anxiety symptoms, improve emotional regulation, and facilitate health behavior change among the sample studied. Despite these promising results, future research should evaluate the pilot findings with larger samples in confirmatory gold standard randomized controlled trials, which will also allow the cross-cultural investigation of the mechanisms of action of MBIs. The extension of MTPC studies to diverse populations would contribute to its dissemination in the healthcare system. Finally, the increasing use of culturally relevant measures related to social connectedness, isolation, and acceptance of mindfulness in the context of faith, in addition to the use of objective health behavior (e.g., accelerometers) and physiologic and neurobiological measures (26, 112), as well as the evaluation of the impact of MBIs on resilience to cope with discrimination-related stressors, would be beneficial (113–115).

## Data Availability Statement

The raw data supporting the conclusions of this article will be made available by the authors, without undue reservation.

## Ethics Statement

The studies involving human participants were reviewed and approved by Cambridge Health Alliance Institutional Review Board. The patients/participants provided their written informed consent to participate in this study.

## Author Contributions

MT: co-designed and executed the study, contributed to the translation of research materials and MTPC cultural adaptation, analyzed the results, and co-wrote the manuscript. TC: analyzed the results, edited the methods and results, and critically reviewed the manuscript for important intellectual content (CRMIIC). MD and NR: co-designed and oversaw the study, contributed to MTPC cultural adaptation, and CRMIIC. LC: executed the study, contributed to the translation of research materials and MTPC cultural adaptation, reviewed data, co-wrote introduction session, and CRMIIC. LS: analyzed the results, edited the methods and results, and CRMIIC. AO: executed the study and co-designed MTPC, contributed to MTPC cultural adaptation, and CRMIIC. AR and NM: executed the study and contributed to the translation of research materials and CRMIIC. MH: contributed to translation of research materials, MTPC cultural adaptation, and CRMIIC. AC: executed the study and CRMIIC. RG: co-designed MTPC, co-designed and oversaw the study, contributed to MTPC cultural adaptation, and CRMIIC. TG: oversaw the study, co-designed MTPC, and CRMIIC. BC: contributed to MTPC cultural adaptation and CRMIIC. ZS-O: co-designed MTPC, co-designed and oversaw the study, contributed to MTPC cultural adaptation, co-wrote the manuscript, and supervised all steps. All authors contributed to the article and approved the submitted version.

## Funding

This study was made possible through grant funding provided by a cooperative agreement supported by the NIH Common Fund Science of Behavior Change Initiative and the National Center for Complementary and Integrative Health: Mindfulness Influences on Self-Regulation: Mental and Physical Health Implications (UH2AT009145/UH3AT009145) (PI: Loucks, Project PI: ZS-O). Additional funding was provided by the Arthur Vining Davis Foundations (PI: ZS-O), the Arnold P. Gold Foundation (PI: ZS-O), Havard Catalyst Disparities Research Award (PI: Desbordes), as well as with funding from Cambridge Health Alliance. MT work was supported by CAPES Brasil–Coordenação de Aperfeiçoamento de Pessoal de Nível Superior under Grant 88882.346691–Finance Code 001 and the Center for Mindfulness and Compassion/CHA.

## Conflict of Interest

The authors declare that the research was conducted in the absence of any commercial or financial relationships that could be construed as a potential conflict of interest.

## Publisher's Note

All claims expressed in this article are solely those of the authors and do not necessarily represent those of their affiliated organizations, or those of the publisher, the editors and the reviewers. Any product that may be evaluated in this article, or claim that may be made by its manufacturer, is not guaranteed or endorsed by the publisher.

## Abbreviations

MT: Marcelo Trombka
TC: Timothy B. Creedon
MD: Marcelo Demarzo
LC: Letícia T. Cuoco
AO: Alexandra C. Oxnard
AR: Alana T. Rozembaque
MH: Marcio S. Hirayama
NM: Natalia B. Moreno
RW: Richa Gawande
TG: Todd Griswold
NR: Neusa S. Rocha
ZSO: Zev Schuman-Olivier.
