# Supplementary material for: Mindfulness Training for Primary Care for Portuguese-Speaking Immigrants: A Pilot Study
Source: Front Psychiatry. 2021 Sep 9;12:664381. doi: 10.3389/fpsyt.2021.664381 (PMC8458702; doi:10.3389/fpsyt.2021.664381)
Supplement: Supplementary file 1 [file Data_Sheet_1.docx]

**STOP-ACHE-GO**

**Stop**or **S**low down

**Turn towards** experience or **Take** notice of breathing

**Observe with Openness**(thoughts, sensations, emotions, urges)
(Diamond of Experience)

**Pleasantness**(notice **P**leasant, un**P**leasant, or neutral feeling tone)

**Allow**it to be as it is,**A**ccept the ACHE is here
or (**A**nchoring to present with breathing)

**Compassion/Curiosity/Common Humanity**

**Hold** the experience with warmth –
bring **H**and to **H**eart or **H**olding touch

**Expand** awareness from ACHE to breathing, then to the body, then to all the senses

&

**Gratitude**(that there is something you can do) then **G**rounding in values

**Open** to life, its challenges and its beauty

**O**rient towards experience with kindness

Copyright © Zev Schuman-Olivier, MD

**MTPC SATISFACTION SURVEY**

**Please indicate how strongly you agree or disagree with the following statements:**

1. I found this program helpful. 1 = Strongly Disagree

2 = Somewhat Disagree 3 = Neutral

4 = Mildly Agree

5 = Strongly Agree

1. The group was well organized. 1 = Strongly Disagree

2 = Somewhat Disagree 3 = Neutral

4 = Mildly Agree

5 = Strongly Agree

1. The group leader(s) cared about me as a person. 1 = Strongly Disagree

2 = Somewhat Disagree 3 = Neutral

4 = Mildly Agree

5 = Strongly Agree

1. I was able to participate and express myself in the 1 = Strongly Disagree group. 2 = Somewhat Disagree

3 = Neutral

4 = Mildly Agree

5 = Strongly Agree

1. The group leader(s) were authentic, honest, and real. 1 = Strongly Disagree

2 = Somewhat Disagree 3 = Neutral

4 = Mildly Agree

5 = Strongly Agree

1. I learned what I was hoping to learn. 1 = Strongly Disagree

2 = Somewhat Disagree 3 = Neutral

4 = Mildly Agree

5 = Strongly Agree

1. The group leader(s) had good timing when providing 1 = Strongly Disagree examples. 2 = Somewhat Disagree

3 = Neutral

4 = Mildly Agree

5 = Strongly Agree

1. I would be willing to participate in the program 1 = Strongly Disagree

again if it were possible to do so. 2 = Somewhat Disagree 3 = Neutral

4 = Mildly Agree

5 = Strongly Agree

1. The group leader(s) were easy to understand. 1 = Strongly Disagree

2 = Somewhat Disagree 3 = Neutral

4 = Mildly Agree

5 = Strongly Agree

1. I would recommend this program to a friend. 1 = Strongly Disagree

2 = Somewhat Disagree 3 = Neutral

4 = Mildly Agree

5 = Strongly Agree

11. Overall rating of the facilitator(s) 1 = Poor 2 = Fair

3 = Good

4 = Very Good 5 = Excellent

12. Overall rating of the program 1 = Poor

2 = Fair

3 = Good

4 = Very Good

5 = Excellent

My favorite part of the program:

The thing I liked least about the program:

The most important thing I learned during this program:

The hardest part of the program:

The most helpful part of the program:

Suggestions for changes in the program:

Table S1. Mixed Effects Analysis – Difficulties in Emotion Regulation Subscales

|  |  |  |  | | | |  | | | | |
| --- | --- | --- | --- | --- | --- | --- | --- | --- | --- | --- | --- |
| **Outcome** | **Baseline** | | **Week 8** | |  | | | **Differences over time** | | | |
|  | **Mean** | **(SE)** | **Mean** | **(SE)** |  | | | ***B*** | **(SE)** | ***p*** | ***d*** |
| **Non-acceptance** | 15.1 | (6.0) | 12.4 | (5.3) | |  | | -2.9 | (1.2) | 0.013* | 0.50 |
| **Goals** | 15.6 | (5.0) | 14.7 | (5.1) | |  | | -1.0 | (1.1) | 0.343 | 0.20 |
| **Impulse** | 12.1 | (4.6) | 11.7 | (5.1) | |  | | -0.5 | (0.9) | 0.548 | 0.11 |
| **Awareness** | 17.7 | (5.9) | 16.0 | (5.5) | |  | | -1.7 | (1.0) | 0.086 | 0.30 |
| **Strategies** | 19.7 | (7.3) | 16.1 | (6.4) | |  | | -3.8 | (1.4) | 0.004* | 0.54 |
| **Clarity** | 11.5 | (4.1) | 10.3 | (3.8) | |  | | -1.4 | (0.8) | 0.065 | 0.36 |

*Significant after Hochberg FDR procedure, family-wise p < 0.05

^ Significant before Hochberg FDR procedure
